# Current Knowledge of Selected Cardiovascular Biomarkers in Pediatrics: Kidney Injury Molecule-1, Salusin-α and -β, Uromodulin, and Adropin

**DOI:** 10.3390/children9010102

**Published:** 2022-01-13

**Authors:** Mirjam Močnik, Nataša Marčun Varda

**Affiliations:** 1 Department of Paediatrics, University Medical Centre Maribor, Ljubljanska 5, 2000 Maribor, Slovenia; natasa.marcunvarda@siol.net; 2 Medical Faculty, University of Maribor, Taborska 8, 2000 Maribor, Slovenia

**Keywords:** kidney injury molecule-1, salusin-α and -β, uromodulin, adropin, cardiovascular biomarkers

## Abstract

Cardiovascular diseases are the leading cause of morbidity and mortality in the modern world. Their common denominator is atherosclerosis, a process beginning in childhood. In pediatrics, the aim of preventive measures is to recognize children and adolescents at risk for accelerated atherosclerosis and possible premature cardiovascular events in adulthood. Several diagnostic procedures and biomarkers are available for cardiovascular risk assessment in adults. However, reliable markers in pediatrics are still insufficiently studied. In this contribution, we discuss five potential biomarkers of particular interest: kidney injury molecule-1, salusin-α and -β, uromodulin, and adropin. Studies regarding the pediatric population are scarce, but they support the evidence from studies in the adult population. These markers might entail both a prognostic and a therapeutic interest.

## 1. Introduction

Cardiovascular diseases, such as myocardial infarction, and cerebrovascular diseases, such as stroke, remain the leading cause of morbidity and mortality in the modern world [1]. Atherosclerosis is a complex, chronic, and multifaceted lifelong process of vascular change, affected by numerous factors, ultimately leading to cardiovascular and cerebrovascular events [2,3]. The first clinical entity of atherosclerosis is endothelial dysfunction, which is an early predictor of subsequent cardiovascular events or mortality [4].

Several classic prognostic factors in cardiovascular disease are being evaluated in order to recognize patients at increased risk for subclinical atherosclerosis and possible premature cardiovascular events. Classic cardiovascular risk factors such as arterial hypertension, hypercholesterolemia, diabetes mellitus, and smoking are associated with endothelial dysfunction. The presence of several risk factors produces synergistic effects on endothelial function as well as the associated cardiovascular prognosis. Cardiovascular risk factors are not rarely present together, also in youths [5]. There is no single marker with ultimate prognostic value. In fact, especially for entities with complex pathogenesis and etiology such as cardiovascular diseases, using and combining several biomarkers might offer several advantages and be beneficial [4,5].

Non-classical indicators of atherosclerosis include markers of the inflammatory process, markers of atherosclerotic plaque injury, acute-phase proteins, ischemic markers, markers of tissue necrosis, and markers of myocardial dysfunction. Their identification enables improved cardiovascular risk profiling [6].

Several new potential biomarkers are being researched with the aim of finding an early footprint, potentially allowing the development of individualized preventive measures [4]. While children with traditional cardiovascular risk factors are already recognized as a category deserving special attention and appropriate interventions [7], there is a need for a better understanding and more detailed, tailored intervention advice among children with “novel”, non-traditional situations of increased cardiovascular risk, such as prematurity, congenital heart diseases, chronic kidney disease (CKD), previous solid organ transplant, chronic inflammatory conditions, sickle cell anemia, and so on. For these, the knowledge of a higher risk according to new biomarkers would be of utmost importance to follow and treat them earlier. Since we do not have an ideal marker of atherosclerosis, nor biochemical, functional, or radiologic markers, the search for reliable and “easy-to-use” atherosclerotic biomarkers in children is still ongoing.

A biomarker is defined as a measure that characterizes, in a quantitative manner, a process of interest. In recent years, process models have much improved properties for extrapolation and prediction [8]. In this paper, we will focus on some specific biomarkers that are being increasingly recognized as potential cardiovascular biomarkers, namely, kidney injury molecule 1 (KIM-1), salusin-α and -β, uromodulin, and adropin, with an emphasis on their prognostic value in pediatric patients. The markers presented were selected arbitrarily and are a prototype of several further possible markers, given the many possible mechanisms involved in the development of atherosclerosis.

## 2. Kidney Injury Molecule 1

### 2.1. What Is It?

**Figure 1:**
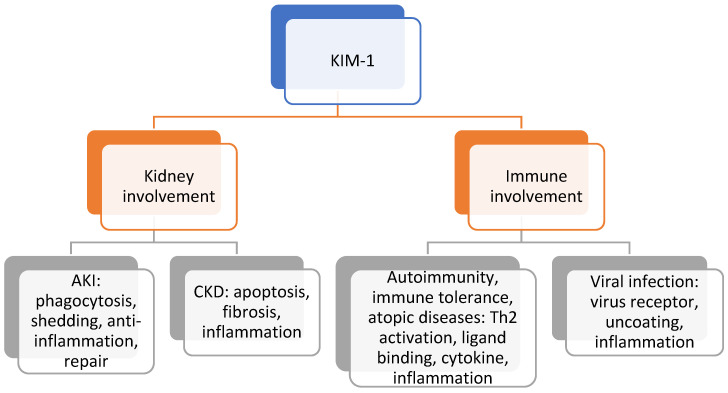
The role of KIM-1 in different diseases with underlying pathophysiologic processes. KIM-1—kidney injury molecule 1, AKI—acute kidney injury, CKD—chronic kidney disease.

KIM-1 is a type I membrane protein, expressed in the kidneys, liver, and spleen, consisting of an extracellular and a cytoplasmic portion. The protein encoded by the KIM-1 gene is a membrane receptor for both human hepatitis A virus and T-cell immunoglobulin mucin domain containing 4. Alternative splicing of this gene results in multiple transcript variants. KIM-1 plays different roles via various molecular targets in immune diseases and kidney injury (Figure 1) [9].

Normal kidneys rarely express KIM-1. However, in some kidney diseases, such as renal tubulointerstitial and polycystic kidney diseases, the expression of KIM-1 is more pronounced because its role is the removal of apoptotic cells and necrotic tissue fragments. The extracellular portion of KIM-1 is cleaved during the process, and its elevated levels can be detected in urine. KIM-1 is also involved in the repair process after injury. The elevation of KIM-1 correlates to the extent of kidney damage. It is generally accepted as an early biomarker of acute kidney injury, but it also has a potential role in predicting long-term renal outcome [9,10].

The value of KIM-1 in acute and chronic settings differs. In the early stage of acute kidney injury, the increased expression of KIM-1 promotes cell phagocytosis, repairs tubular injury, and inhibits the renal inflammatory response. In contrast, KIM-1 has been shown as a biomarker for chronic proximal tubular injury, where higher levels of KIM-1 correlate with the occurrence and development of renal fibrosis [10]. Urinary samples of KIM-1 correlate significantly with the incidence and prognosis of chronic kidney disease [11,12,13], and in more severe chronic kidney disease, KIM-1 was found to be an independent risk factor for progression to end-stage renal disease [14]. Additionally, its role in inflammation was evident in the progression of IgA nephropathy leading to more serious and rapid disease progression with higher levels of KIM-1 [15]. Similarly, increased levels of KIM-1 were associated with inflammation of the renal tubules in protein-overload nephropathy in animal studies [16].

### 2.2. How Is It Involved in Cardiovascular Risk?

Chronic kidney disease and cardiovascular diseases are closely associated, and the dysfunction of either can sometimes be referred to as cardiorenal syndrome. Five subtypes have been proposed: type 1, acute cardiorenal syndrome (acute impairment of heart function leads to kidney damage); type 2, chronic cardiorenal syndrome (chronic heart diseases lead to kidney damage); type 3, acute renocardiac syndrome (acute kidney damage leads to heart injury); type 4, chronic renocardiac syndrome (chronic kidney disease leads to heart disease); and type 5, secondary cardiorenal syndrome occurring in systemic disorders (e.g., sepsis, diabetes mellitus, and amyloidosis) simultaneously causing both cardiac and renal dysfunctions [17,18]. Its pathophysiology includes reduced renal perfusion, increased venous pressure, and the activation of multiple neurohormonal systems; however, the whole process is still not completely understood. KIM-1 was confirmed as an excellent prognostic marker for the detection of acute tubular injury in patients with chronic heart failure and showed better prognostic role in how changes in volume status can lead to subclinical tubular injury, undetected by traditional biomarkers, such as creatinine, GFR, cystatin C, and proteinuria [17].

KIM-1 was associated with increased risk of death or hospitalization, independent of glomerular filtration rate (GFR), in patients with chronic heart failure [19]. It was also elevated in symptomatic heart failure in patients with apparently normal kidney function, indicating tubular injury in chronic heart failure [20]. KIM-1 was found to be an effective clinical biomarker for hypertension associated with CKD [21]. In the elderly, higher KIM-1 was associated with a higher risk of cardiovascular mortality independently of the established cardiovascular risk factors, GFR and albuminuria [22]. It was also found as one of the important protein biomarkers for major adverse cardiovascular events in adult diabetic patients [23].

### 2.3. What Is Its Role in Cardiovascular Risk in Pediatric Patients?

In contrast to adults, there is controversy regarding the role of KIM-1 in the prediction of acute kidney injury in children, and it seems to have a moderate prognostic value [24], having potential in certain settings such as kidney damage detection after chemotherapy [25]. However, KIM-1 was also found to be associated with CKD including tubular injury and inflammation in the pediatric population, indicating its potential role in evaluating CKD and cardiovascular risk [26]. Additionally, as compared to healthy children, KIM-1 was higher in normoalbuminuric diabetic children before reduction in GFR [27], and even in children with vesicoureteral reflux [28]. Obesity, the most prominent factor of cardiovascular risk in children, has been associated with higher KIM-1 levels in children [29,30], suggesting a possible effect of obesity in kidney tubular damage; however, not all studies confirmed this finding [31]. Therefore, the role of KIM-1 in cardiovascular risk in children is not conclusive [24,31]. Further research in the field is needed to understand how KIM-1 could be used as cardiovascular biomarker in children.

## 3. Salusin-α and -β

### 3.1. What Are They?

**Figure 2:**
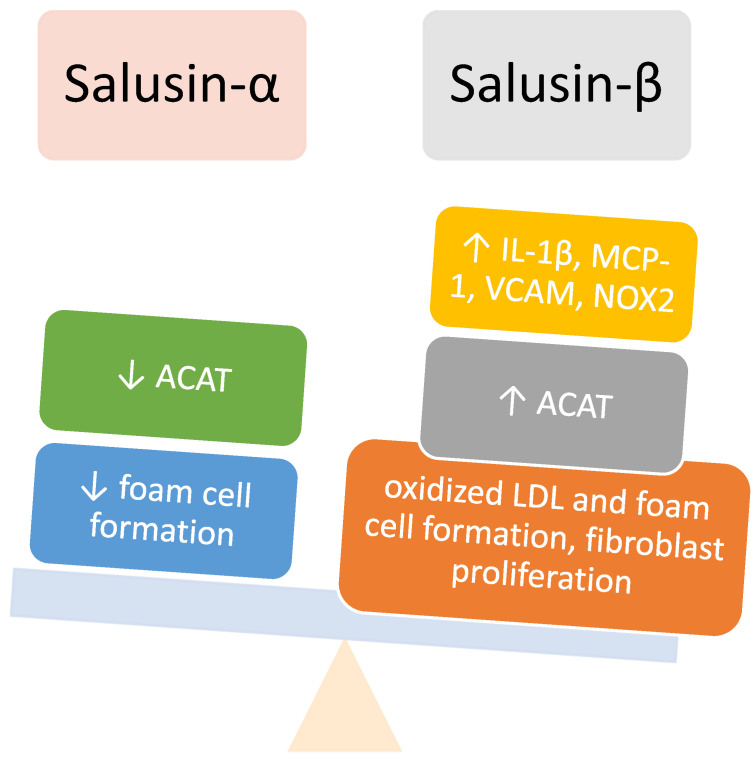
Modulatory effects of salusin-α and -β. ACAT—acyl-CoA: cholesterol acyltransferase 1; IL-1β—interleukin-1β, MCP—monocyte chemoattractant protein-1; VCAM—vascular cell adhesion molecules; NOX2—NADPH oxidase 2.

Salusin-α and -β are endogenous proteins that seem to play a significant role in hemodynamics. They are present in human plasma and urine, indicating their possible role as peptide hormones. They belong to a new class of peptides discovered by bioinformatics technology and are considered to be synthesized from preprosalusin, an alternative splicing product of the torsion dystonia-related gene. They are expressed in many tissues in the body, and, importantly, also in atherosclerotic plaques [32,33]. The modulatory effects of salusin-α and -β are schematically shown in Figure 2.

Salusin-α and -β induce opposite effects on foam cell formation. They act on down-regulation (salusin-α) and up-regulation (salusin-β) of acyl-coenzyme A: cholesterol acyltransferase-1, an enzyme stimulating the accumulation of cholesterol esters in macrophages [34]. Additionally, their mitogenic activity, especially of salusin-β, affecting vascular cells, has been researched. Salusin-β stimulates the proliferation of vascular smooth muscle cells and vascular fibrosis. Its expression is pronounced in neointimal hyperplasia after coronary stent implantation [35]. Therefore, salusin-β promotes and salusin-α reduces atherosclerosis. The multifunctional nature of salusins in the cardiovascular system was demonstrated by the induction of hypotension and bradycardia, as well as the decrease in cardiac contractility after salusin-β infusion [36]. However, their function probably exceeds the cardiovascular system due to their expression in many human tissues, including those of the small intestine, stomach, adrenal medulla, thymus, lymph nodes, spleen, bone marrow, salivary glands, lungs, skeletal muscle, testes, adrenal cortex, brain, and liver [37].

### 3.2. How Are They Involved in Cardiovascular Risk?

According to their function, they are believed to be associated with atherosclerosis and cardiovascular diseases. Hypertensive patients demonstrated lower levels of salusin-α with an additional negative correlation to brachial-ankle pulse wave velocity and intima media thickness [38,39]. Additionally, non-dipper hypertension patients had lower salusin-α and higher salusin-β levels compared to those with dipper hypertension. In the same study, both salusins were also associated with a left ventricular mass [40]. In patients with coronary atherosclerosis, salusin-α and, to a greater extent, salusin-β were associated with the degree of coronary stenosis [41,42].

Due to their action, involvement in lipid metabolism is expected. Nazari et al. published a study regarding women with overweight or obesity, where there was a significant increase in salusin-α and high-density lipoprotein levels with moderate- and high-intensity interval training groups. At the same time, a significant decrease in triglycerides and total cholesterol was observed. There were also insignificant reductions in salusin-β, low-density lipoprotein, and very-low-density lipoprotein levels [43].

Additionally, salusins appear to be modified in patients with type 2 diabetes mellitus with significantly higher levels of salusin-β and lower levels of salusin-α among diabetics compared to healthy controls. Salusin-β correlated positively with fasting glucose and glycated hemoglobin, while salusin-α correlated negatively with the same parameters. There were no associations between salusins and obesity [44].

### 3.3. What Are Their Roles in Cardiovascular Risk in Pediatric Patients?

Studies regarding salusins in the pediatric population are scarce, but existing data support the findings of the adult population. Similarly, the concentration of salusin-β was higher in pediatric patients with essential hypertension. Additionally, it correlated positively with the body mass index Z-score and triglyceride levels [45,46]. In another study, salusin-α correlated negatively with diastolic blood pressure; however, no significant differences were found between normal-weight and overweight children [47]. Similarly, both of the salusins are useful in training effectiveness, demonstrating a significant improvement of both markers in aerobic and high-intensity interval training [48].

## 4. Uromodulin

### 4.1. What Is It?

Uromodulin is a glycoprotein that is synthesized exclusively in the kidney in epithelial cells in the thick ascending part of the loop of Henle from the UMOD gene. It is the most abundant protein in normal urine. Its function is immunomodulatory with protection against urinary tract infections. Upon contact with bacteria, it participates in the activation of granulocytes and monocytes and inhibits the proliferation of T lymphocytes; it therefore acts as a pro- and anti-inflammatory factor. Uromodulin is cleaved at the apical membrane and released in the urine where it forms macromolecular polymers [49,50].

**Figure 3:**
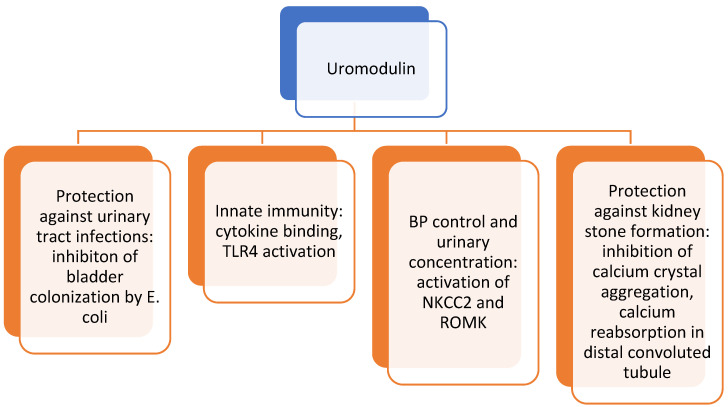
Proposed physiological roles of uromodulin. TLR4—Toll-like receptor 4; NKCC2—Na+-K+-2Cl-cotransporter; ROMK—potassium channel ROMK, BP—blood pressure.

Uromodulin was discovered decades ago, but its mechanism of action remained elusive. Recent in vitro studies showed that uromodulin inhibits viral hemagglutination and suppresses antigen-mediated T-cell proliferation and monocyte function. Through specific cell surface receptors, it helps to regulate chemotaxis, phagocytosis and apoptosis, and facilitates neutrophil trans-epithelial migration. Additional studies observed that uromodulin forms a hydrophobic, gel-like structure that led to the suggestion of its action as a seal that contributes to the water impermeability of thick ascending tubules. Furthermore, it was shown that uromodulin regulates the activity of Na+-K+-2Cl-cotransporter NKCC2 and the potassium channel ROMK, both influencing the tonicity of the medulla and urinary concentrating ability. The impaired function of both channels led to reduced renin biosynthesis. In humans, a high salt intake also led to increased excretion of uromodulin, and the opposite was true for a low salt intake. In animal studies, the overexpression of uromodulin led to salt-sensitive hypertension and an increased response to furosemide [50,51,52]. The proposed physiological roles of uromodulin are presented in Figure 3.

Uromodulin is negatively charged in the urine, which inhibits crystal aggregation. Additionally, the capacity of uromodulin to modulate the activity of thick ascending tubules might have an impact on urinary concentration and on the paracellular handling of Ca^2+^, thereby influencing the urinary concentration of solutes in the urine [48,50]. On the other hand, uromodulin can act as a part of hyaline cast leading to low-molecular-weight proteinuria and cast nephropathy [50,52].

Uromodulin is found in both urine and serum. Urinary uromodulin has been studied in patients with CKD, where its levels were correlated with GFR. Higher uromodulin levels were present before CKD development and were associated with the development of end-stage renal disease [49,50,51,52].

### 4.2. How Is It Involved in Cardiovascular Risk?

According to its function, uromodulin has been regarded for a long time primarily as a kidney function marker with prognostic value in some congenital kidney diseases, tubular function, GFR estimation, kidney stones, and acute kidney injury. However, its role in cardiovascular risk is important from several points of view. The first is the above-mentioned connection to cardiorenal syndrome. The second reason is the association between uromodulin and salt-sensitive hypertension. More specifically, uromodulin regulates sodium uptake in the thick ascending limb of the loop of Henle by modulating the effect of tumor necrosis factor-α on NKCC2A expression, making UMOD an important determinant of blood pressure control and a candidate gene for essential hypertension [53]. Additionally, some variants in the uromodulin gene promoter are associated with CKD and hypertension [50,54], and some variants with left atrial remodeling [55].

In the prognosis of cardiovascular diseases, urinary and serum uromodulin was associated with cardiovascular risk and events. Lower urinary uromodulin levels were associated with patients at risk of progressive kidney disease and mortality [56]. When the urinary and serum uromodulin levels were compared, their correlates differed substantially, suggesting that apical and basolateral secretion might be differentially regulated. Urinary uromodulin is more strongly associated with GFR [57]; however, serum uromodulin has a better prognostic value in cardiovascular risk. Higher serum uromodulin was associated with a favorable metabolic profile, lower prevalence rates of comorbidities, and lower risk of kidney failure and overall 10-year mortality, independent of other cardiovascular risk factors [58,59,60,61].

### 4.3. What Is Its Role in Cardiovascular Risk in Pediatric Patients?

In the pediatric population, the marker is still largely unexplored. Its role is promising in the prediction of acute kidney injury and may also be used as a prognostic biomarker for recovery from acute kidney injury [62,63]. A difference was found in children with type 1 diabetes, where the patients had lower serum uromodulin levels compared to healthy patients, and, at the same time, the level was negatively correlated with microalbuminuria [64]; however, for other patient groups in the pediatric population, the research is still ongoing. For cardiovascular risk, the prognostic value of uromodulin has not yet been evaluated in children.

## 5. Adropin

### 5.1. What Is It?

Adropin is a small peptide encoded by the energy homeostasis-associated gene ENHO, involved in glucose homeostasis and lipid metabolism [33,65]. It is expressed mainly in the liver and brain with a presumed short half-life. Recently, it was discovered that adropin is important for energy homeostasis, lipid metabolism, and the maintenance of insulin sensitivity. Animal studies demonstrated improved glucose homeostasis, fatty liver, and dyslipidemia after adropin infusion, which indicates its potential role in therapy. Moreover, the level of adropin in animal studies was associated with the amount of fat intake, indicating that its expression is regulated by feeding and suppressed by fasting [33,65,66]. Adropin presumably inhibits fat oxidation by suppressing the expression and activity of carnitine palmitoyltransferase-1b [66].

**Figure 4:**
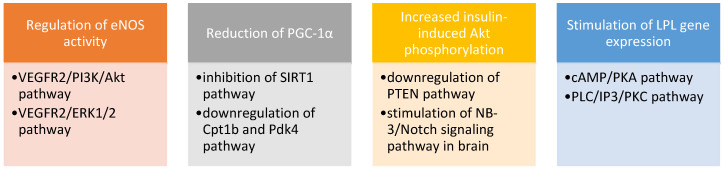
Adropin-triggered intracellular signal transduction pathways. eNOS—endothelial nitric oxide synthase; VEGFR2—vascular endothelial growth factor receptor 2; PI3K—phosphatidylinositol 3-kinase; Akt—protein kinase B; ERK1/2—extracellular signal-regulated kinase ½; PGC-1α—peroxisome proliferators-activated receptor-γ coactivator-1α; SIRT1—silent information regulator 1; Cpt1b—carnitine palmitoyltransferase 1B; Pdk4—pyruvate dehydrogenase kinase; PTEN—phosphatase and tensin homolog deleted on chromosome ten; LPL—lipoprotein lipase; NB-3—neural recognition molecule 3; cAMP—cyclic adenosine monophosphate; PKA—protein kinase A; PLC—phospholipase C; IP3—inositol trisphosphate; PKC—protein kinase C.

In vitro, adropin-treated endothelial cells also demonstrated greater proliferation and migration. Research indicates that adropin could exert its impact on cell functions by up-regulation of endothelial nitric oxide synthase. It is also expressed in human coronary artery endothelial cells [33]. Its involvement has been shown in many intracellular signal transduction pathways, presented in Figure 4.

### 5.2. How Is It Involved in Cardiovascular Risk?

In humans, lower levels of adropin are associated with several classical cardiovascular or metabolic risk factors, which are related to cardiovascular diseases. Additionally, its action on endothelial dysfunction indicates its direct involvement in atherosclerosis development [33]. Circulating adropin levels may therefore serve as an early biomarker by which to predict the development of endothelial dysfunction before the emergence of clinical symptoms of specific patient groups.

Patients with ischemic heart disease had lower adropin levels, with even lower levels in patients with myocardial infarction [33,67,68]. Similarly, adropin levels were lower in patients with occluded saphenous vein grafts, used in coronary artery bypass grafting, than in those with patent grafts [69]. It has been shown that during myocardial injury, adropin synthesis increased, and serum adropin levels were elevated as early as one hour post-infarction, indicating its possible role in myocardial infarction diagnosis as an alternative to troponin I [70]. Being associated with vasculature, the levels of adropin in patients with erectile dysfunction also correlated to the index of the erectile function questionnaire [71]. Lower circulating adropin levels were closely associated with endothelial dysfunction in patients with obstructive sleep apnea–hypopnea syndrome [72].

The primary role of adropin, according to animal studies, seems to be in lipid and glucose metabolism. The overexpression of adropin led to reduced weight gain and lower fasting levels of insulin and triglycerides [73]. Furthermore, in animal studies, it has been demonstrated that energy expenditure is also affected by adropin in the heart and may present a treatment option for patients with cardiac disease associated with insulin sensitivity [74,75]. In fact, adropin administration in animal models led to enhanced glucose tolerance, amelioration of insulin resistance, and promotion of glucose preference while suppressing fat oxidation [76]. In humans, adropin levels were significantly lower in type 2 diabetic patients and were associated with angiographic severity of coronary atherosclerosis [77]. Additionally, in patients undergoing hemodialysis, adropin was found as a useful marker of cardiac dysfunction with non-significant changes in its concentration during hemodialysis [78].

### 5.3. What Is Its Role in Cardiovascular Risk in Pediatric Patients?

Some studies have been conducted in children that support the findings from animal studies and the adult population; however, the data are scarce, and more research is needed in this area. Similar to the adult population, adropin in children seems to be involved in energy homeostasis, lipid metabolism, and the maintenance of insulin sensitivity. Therefore, increased levels of adropin could affect impaired glucose and lipid metabolism, exposing a child to greater metabolic cardiovascular risk. It has been demonstrated that obese children have lower levels of adropin [79,80]. Adropin levels also increased in obese children after an exercise intervention [81]. In addition, lower adropin levels were an independent risk factor for non-alcoholic fatty liver disease in obese adolescents [79]. However, no correlation between serum adropin and blood pressure was observed [80]. Interestingly, adropin levels could be used as an auxiliary biomarker in Kawasaki disease. It has been shown that adropin levels were significantly higher in children with Kawasaki disease, and even higher in children with coronary artery lesions due to Kawasaki disease [82]. Adropin levels were also reduced in children with obstructive sleep apnea when endothelial dysfunction was present and returned to within normal values after adenotonsillectomy [83].

## 6. Other Investigated Potential Cardiovascular Biomarkers

Several other biomarkers of cardiovascular risk are emerging, including biomarkers representing regulators of metabolic homeostasis and inflammatory pathways. New discoveries may be informative for causal biological pathways contributing to disease, allowing the potential for pathway-specific therapies and personalized treatments. The discovery of novel prognostic biomarkers of cardiovascular risk has the potential to improve risk assessment in the preclinical phase of the disease, when intervention is most likely to be effective. In adults, several additional biomarkers are being investigated, such as growth differentiation factor 15 (GDF15), tissue inhibitor of metalloproteinase-1 (TIMP1), beta-2-microglobulin (B2M), adrenomedullin (ADM), C-type lectin domain family 3 member B (CLEC3B), insulin-like growth factor 1 (IGF1), butyrylcholinesterase (BCHE), paraoxonase 1 (PON1), insulin-like growth factor binding protein 1 (IGFBP3), insulin-like growth factor binding protein 2 (IGFBP2), contactin-1 (CNTN1), kallikrein B1 (KLKB1), peripheral myelin protein 2 (PMP2), arabinogalactan protein 1 (AGP1), soluble receptor for advanced glycation end products (sRAGE), uncarboxylated matrix Gla protein (UCMGP), and matrix metalloproteinase 9 (MMP9), etc. [84]. There are numerous studies being conducted that aim to predict myocardial infarction in adults; however, in children, recent studies are focused on the prediction of early cardiovascular disease in obese children [85,86,87,88]. Along with the presented markers, other investigated most often include resistin, leptin, myeloperoxidase (MPO), total plasminogen activator inhibitor 1 (TPAI-1) [84], adiponectin, interleukin 6 (IL-6) [86], and other inflammatory markers such as interleukin 10 (IL-10), tumor necrosis factor alpha (TNF-α), and thrombomodulin, etc.

Additionally, microribonucleic acids (miRNAs), non-coding RNAs, are being investigated with lightning speed. Their involvement in the cardiovascular system has been shown through their basic functions in all cell types relevant to the cardiovascular system (endothelial cells, cardiac muscle, smooth muscle, inflammatory cells, and fibroblasts), and they are therefore directly involved in the development of cardiovascular diseases. Their use is being studied in diagnostics, prognostics, and therapy [87]. Some of the miRNAs have also been identified in children with obesity and non-alcoholic fatty liver disease, presenting a promising future diagnostic and prognostic tool [88].

The search for reliable markers that might work together, thereby allowing the creation of a “cardiovascular risk footprint”, is important in the pediatric population. This may allow the identification of individuals at increased early cardiovascular risk, and would therefore allow the timely planning of preventive and therapeutic interventions. We still do not know to what degree an individual child is prone to premature cardiovascular events in adult life. With new biomarkers, the search is ongoing to identify children at risk for accelerated atherosclerosis and premature cardiovascular and cerebrovascular events. Future research should ideally include different groups of children with and without cardiovascular risk factors and the testing a set of markers of purported pathophysiological mechanisms. These might include inflammatory mediators and mediators of endothelial dysfunction and of oxidative stress. Further, early markers of atherosclerosis, such as intima media thickness and pulse wave velocity, should also be additionally investigated. Globally, this cumulative knowledge might contribute to unravelling potential therapeutic and preventive interventions.

## 7. Conclusions

Cardiovascular diseases are still the leading cause of morbidity and mortality in modern society. Several new potential biomarkers are being investigated and evaluated. We arbitrarily selected five potentially interesting biomarkers, but many more are under investigation. KIM-1 and uromodulin are classical kidney function and damage markers that are being increasingly evaluated in the context of cardiovascular risk. Salusins and adropin are new proteins that are emerging as markers of cardiovascular risk in adults, but less is known about their value in the pediatric population. These markers have a potential role in cardiovascular diagnostics and risk assessment and should be further researched in clinical practice.

## Data Availability

All the data are available within the article.
